# Prediction of Patient Satisfaction after Treatment of Chronic Neck Pain with Mulligan’s Mobilization

**DOI:** 10.3390/life13010048

**Published:** 2022-12-23

**Authors:** Josué Fernández-Carnero, Hector Beltrán-Alacreu, Alberto Arribas-Romano, Ester Cerezo-Téllez, Juan Nicolás Cuenca-Zaldivar, Eleuterio A. Sánchez-Romero, Sergio Lerma Lara, Jorge Hugo Villafañe

**Affiliations:** 1 Department of Physical Therapy, Occupational Therapy, Rehabilitation and Physical Medicine, Universidad Rey Juan Carlos, 28922 Alcorcón, Spain; 2 Musculoskeletal Pain and Motor Control Research Group, Faculty of Sport Sciences, Universidad Europea de Madrid, 28670 Madrid, Spain; 3 Department of Physiotherapy, Faculty of Sport Sciences, Universidad Europea de Madrid, 28670 Villaviciosa de Odón, Spain; 4 Musculoskeletal Pain and Motor Control Research Group, Faculty of Health Sciences, Universidad Europea de Canarias, C/Inocencio García 1, 38300 La Orotava, Spain; 5 Department of Physiotherapy, Faculty of Health Sciences, Universidad Europea de Canarias, 38300 Santa Cruz de Tenerife, Spain; 6 Motion in Brains Research Group, Institute of Neuroscience and Sciences of the Movement (INCIMOV), Centro Superior de Estudios Universitarios La Salle, Universidad Autónoma de Madrid, 28023 Madrid, Spain; 7 Toledo Physiotherapy Research Group (GIFTO), Faculty of Physical Therapy and Nursing, Universidad de Castilla-La Mancha, Avenida de Carlos III s/n, 45071 Toledo, Spain; 8 CranioSPain Research Group, Centro Superior de Estudios Universitarios La Salle, Calle de la Salle 10, 28023 Madrid, Spain; 9 International Doctoral School, Rey Juan Carlos University, 28933 Móstoles, Spain; 10 Facultad de Medicina y Ciencias de la Salud, Departamento de Enfermería y Fisioterapia, Grupo de Investigación en Fisioterapia y Dolor, Universidad de Alcalá, 28801 Alcalá de Henares, Spain; 11 Research Group in Nursing and Health Care, Puerta de Hierro Health Research Institute-Segovia de Arana (IDIPHISA), Manuel de Falla s/n, 28220 Majadahonda, Spain; 12 Primary Health Center “El Abajón”, Calle Principado de Asturias 30, 28231 Las Rozas, Spain; 13 Department of Physical Therapy, Centro Superior de Estudios Universitarios La Salle, Universidad Autónoma de Madrid, 28023 Madrid, Spain; 14 IRCCS Fondazione Don Carlo Gnocchi, Piazzale Morandi 6, 20148 Milan, Italy

**Keywords:** chronic neck pain, musculoskeletal manipulations, machine learning, Mulligan concept, manual therapy, chronic pain

## Abstract

Chronic neck pain is among the most common types of musculoskeletal pain. Manual therapy has been shown to have positive effects on this type of pain, but there are not yet many predictive models for determining how best to apply manual therapy to the different subtypes of neck pain. The aim of this study is to develop a predictive learning approach to determine which basal outcome could give a prognostic value (Global Rating of Change, GRoC scale) for Mulligan’s mobilization technique and to identify the most important predictive factors for recovery in chronic neck pain subjects in four key areas: the number of treatments, time of treatment, reduction of pain, and range of motion (ROM) increase. A prospective cohort dataset of 80 participants with chronic neck pain diagnosed by their family doctor was analyzed. Logistic regression and machine learning modeling techniques (Generalized Boosted Models, Support Vector Machine, Kernel, Classsification and Decision Trees, Random Forest and Neural Networks) were each used to form a prognostic model for each of the nine outcomes obtained before and after intervention: disability—neck disability index (NDI), patient satisfaction (GRoC), quality of life (12-Item Short Form Survey, SF-12), State-Trait Anxiety Inventory (STAI), Beck Depression Inventory (BDI II), pain catastrophizing scale (ECD), kinesiophobia-Tampa scale of kinesiophobia (TSK-11), Pain Intensity Visual Analogue Scale (VAS), and cervical ROM. Pain descriptions from the subjects and pain body diagrams guided the physical examination. The most important predictive factors for recovery in chronic neck pain patients indicated that the more anxiety and the lower the ROM of lateroflexion, the higher the probability of success with the Mulligan concept treatment.

## 1. Introduction

Chronic neck pain is a highly prevalent disorder in primary healthcare services presenting a prevalence from 5.9% to 38.7% in the general population [1,2,3,4], 19.5% in Spain [5,6], and 13.8% in the USA [5]. This pathology represents 0.6% of the world population who suffers from disabling neck pain [7], which is 18% of the population [8], and it causes 10% of job absenteeism [9,10,11,12] and disability [7,9] in the long term. It also involves significant health and socioeconomic problem in industrialized countries [10].

In this sense, most of the cases of acute neck pain will be resolved with or without treatment, while 50% of these cases will continue suffering from neck pain, affecting their quality of life [10] and function. Sedentary life, spent sitting in front of screens for hours with static positions of the head and shoulders, as workers or students do, produces muscular stress and imbalance and has been described as a cause of chronic neck pain [11,12,13,14]. In a recent study analyzing musculoskeletal rehabilitation needs while taking into account the proportion of prevalent cases and the years lived with disability, neck pain ranked 5th after lower back pain, fractures, osteoarthritis, and other injuries as the condition with the greatest need for musculoskeletal rehabilitation worldwide in the last 3 decades [15]. The evidence for alternative treatments for neck pain, including massage, acupuncture, manipulation, soft cervical collar, electrotherapy, trigger point injections or botulinum injections, and yoga, being superior to sham or other treatments is weak [16]. The evidence for pharmacologic interventions for chronic musculoskeletal neck pain is limited (muscle relaxants, epidural corticosteroids injections, neither NSAIDs nor steroids in oral application) [16]. Topical NSAIDs show low efficacy too. Combined treatment utilizing the pharmacological approach plus conservative treatment was superior to either treatment alone. Neither drug is better than non-pharmacological alternative treatments [17].

Current evidence suggests that manipulation and mobilization are effective therapies for mechanical neck pain [14,18,19], while the McKenzie physical therapy approach [20], dry needling [21], and new approaches such as O_2_O_3_ therapy remain promising conservative methods to improve pain symptoms in these patients [20,22]. As a result, manipulation and mobilization are both frequently used in neck pain treatment and have shown short- and long-term mechanical effects on connective tissue [23] in addition to neurophysiological effects including analgesic, motor, and sympathetic nervous system effects [24]. Cervical manipulation is more effective in reducing pain and increasing the cervical joint range immediately than mobilization combined with soft tissue manipulation techniques [25]. In this sense, Mulligan’s concept consists of performing specific mobilizations at the cervical spine then adding accessory movements, thereby promoting pain-free active physiological movement levels [18].

While there are treatments that seem to be effective in reducing pain for this patient group, [21,26,27] there is a tool that provides a prognostic model identifying the most influential predictors that could either inform the clinical approach or lead to the development of new therapeutic interventions [28] which has shown its reliability in making decisions [29].

Machine learning (ML) refers to a set of techniques in which an algorithm makes predictions to interpret data and “learn” without any static program of instructions; in its supervised form, the algorithm is trained with a set of data that is analyzed for important features for classification and labelling. Before the “trained” model is applied to a new data set [30,31], multiple classification algorithms can be formed, from traditional logistic regression to new approaches like neural networks.

Nowadays, researchers are focusing on ML techniques (using artificial intelligence to enable computers to independently initiate and execute new data) with built-in predictor selection functionality [28] not only for the development of accurate models (for example as few predictors as possible to achieve great predictive accuracy) [28,29,32,33,34] but also to develop new interventions [35]. This method has been widely used in medicine in intensive care units [29], to assess cardiovascular risk at a hospital [33], and even in primary care units as prognostics for headaches [34]. In physical therapy, ML techniques for prognostic modeling have not routinely been used in musculoskeletal pain research, including that of chronic neck pain, thus far, but this is a growing field of interest [36]. However, it has been increasingly developed in other areas such as lumbar pain [37,38] or cervical radiculopathy [28,39]. There is a need to invest time and resources to include all the data into the system, and after, analysis to develop the model.

ML is a tool that has shown potential in assisting clinical decision-making in the field of physiotherapy [40,41], and the development of validated prognosis models represents a growing field [42] that can be cost-efficient, allowing physiotherapists to make predictions and improve rehabilitation performance [43]. Meanwhile, its value is huge, reducing costs and helping health professionals to succeed without wasting resources.

The lack of knowledge in this field is added to by the fact that current predictive studies for neck pain have focused on self-completed predictors [28,44] or objective evaluations [29]. The development of a predictive model that includes both self-completed surveys and physical evaluations could give rise to a valid model, where the number of predictors exceeds the sample size, could not be estimated statistically with traditional methods, and an ML approach would be needed.

Therefore, the aim of this study was to develop a predictive ML approach to determine patient satisfaction after treatment following Mulligan’s mobilization techniques and to identify the most important predictive factors for recovery in chronic neck pain patients.

## 2. Materials and Methods

### 2.1. Design

An experimental prospective cohort study was carried out between August 2021 and March 2022 at the Rey Juan Carlos University Clinic (Alcorcón, Madrid, Spain). The research project was approved by the regional ethics committee in Alcorcon, Madrid, Spain (protocol number 1211202121521). The project was also registered in CTs (NCT05004467). The transparent reporting of a multivariable prediction model for individual prognosis (TRIPOD) guidelines [35] for all methods were used [28].

### 2.2. Sample

A total of 80 subjects aged between 18 and 60 years presented chronic neck pain (without radiation to upper limbs) for a duration of at least 6 months—neck disability index (NDI) values ≥10. Each participant underwent a standardized clinical physiotherapy examination of the neck and upper extremities. Eligible participants gave written informed consent before participation in the study and after being diagnosed with chronic pain by their primary care doctor.

All subjects who filled out the global rating of change scale (GRoC) were assessed and received Mulligan’s neck mobilizations [18]. A physical therapist performed the initial and follow-up assessments of all participants and performed the treatment.

The established exclusion criteria were as follows: any red flag diagnosed by the primary care doctor; major trauma or whiplash in the last 2 years (documented from the medical history); pregnancy; widespread pain [45]; vestibular disorders; having worn a cervical collar in the last year; inflammatory articular illness; neurological disorders affecting the central nervous system; protrusion or hernia; previous surgical intervention of the shoulders, neck, upper limbs, or tendinopathy in the last year; having received any physical therapy treatment of the cervical or thoracic region in the lasts 3 months; tendinopathy in the upper extremities; severe psychiatric illness; withdrawal from the study; or if the subject was unable to speak or write Spanish sufficiently to complete the questionnaires.

In addition, participants were excluded if they were using anti-inflammatory, analgesic, anticoagulant, muscle relaxant, or antidepressant medication one week before the study commenced, had fibromyalgia syndrome, or had any contraindication to conservative or invasive physiotherapy (infection, fever, cancer, or systemic disease).

### 2.3. Data Collection

Data collection was performed by a trained evaluator physical therapist (with more than 10 years of professional experience) using the global rating of change (GRoC), visual analogue scale (VAS), and inclinometer. A validated Spanish version of every questionnaire was used. These questionnaires and scales have already been demonstrated to be reproducible and valid for measuring subjective pain [46], quality of life [47], disability [48], anxiety [49], depression [50], catastrophism [51], kinesiophobia [47], and active mobility [52]. This information was included in an ACCESS document using a double verification system. If during the revision of the data an error in the values or a difference of 5% in any measurement has been detected, this subject was excluded. All scales and questionnaires were completed without any loss of values for their analyses.

### 2.4. Neck Disability Index

This index measures the level of perceived neck disability [53]. It is a self-reporting instrument for the assessment of the condition-specific functional status of subjects with neck pain and includes 10 items: pain, lifting, personal care, reading, concentration, headaches, work, recreation, driving, and sleeping. It scores on a 0 to 5 rating scale (0 meaning ”no pain” and 5 meaning ”worst imaginable pain”). In the end, the sum of all of the subscales results in a score (50 points maximum) or percentage (100%). The NDI has been shown to have a high degree of test–retest reliability, internal consistency, and an acceptable level of validity, being sensitive to changes over time [54].

### 2.5. Patient Satisfaction

The global change evaluation (GRoC) was used to assess patient satisfaction and was considered as the principal variable. This questionnaire has been used in previous studies on patients with neck pain [55,56]. The GRoC is a 15-point scale with a central value of 0 (no change) and values above or to the left ranging from –1 to –7 (much worse), and below or to the right from +1 to +7 (much better) [57,58]. In this study, we consider patients who rated “considerably better” or better (+5 or more) to be those who received successful treatment. It has previously been published that a score of +4 (moderately better) is a suggested cut-off point for dichotomizing improvement versus non-improvement [59].

### 2.6. SF 12 Questionnaire (Standard Version)

Mental health status was used to learn the beliefs of the subjects about their general health and to know whether they are able to conduct their daily life normally [47]. It is considered reliable and valid [60].

### 2.7. State-Trait Anxiety Inventory (STAI)

The STAI questions of this questionnaire are aimed at discovering how the patient feels at that moment [61]. The Spanish version of this questionnaire has acceptable psychometric properties (αstate = 0.94; r = 0.62) [62].

### 2.8. Beck Depression Inventory (BDI II)

The BECK items mainly measure depressive symptoms, specific thoughts of guilt or feelings of punishment, and symptoms such as fatigue or decreased appetite (Beck et al., 1988; Heredia Lima et al., n.d.). This questionnaire has an excellent reliability coefficient (r = 0.92) and a good internal consistency (α = 0.87) [50,63,64,65].

### 2.9. Catastrophism Scale (ECD)

The Spanish version of the pain catastrophizing scale is valid and reliable [35]. It is composed of 13 items considering the aspects of rumination, magnification, and helplessness. Higher scores are associated with greater catastrophism towards pain. The smallest detectable change is identified as 9.1 points [51,66].

### 2.10. Kinesiophobia

The Spanish version of the Tampa scale of kinesiophobia (TSK-11) is used to measure the patient’s degree of fear of movement and has shown good validity and reliability. It consists of 11 questions about the fear of pain during various activities. The lower the score, the lower the fear of movement [67].

### 2.11. Pain Intensity Visual Analogue Scale

This scale was used to measure the actual subjective pain intensity, the maximum and minimum pain felt in the last 24 h, and pre- and post-intervention mechanical pain: rest, flexion, extension, left and right rotation, and inclination [68]. The subjects reported their current pain intensity using a 100 mm VAS, consisting of a 100 mm horizontal line with pain descriptors marked “no pain” on the left side and “the worst imaginable pain” on the right side [69] VAS has already shown its reproducibility and validity of pain intensity [46]. The visual analogue scale has demonstrated high validity (ICC = 0.97), even above other scales such as the numerical scale or the verbal scale [70].

### 2.12. Neck Active Range of Motion

The active range of motion of neck flexion–extension, left and right rotation, and side-bending were recorded using a cervical bubble inclinometer BASELINE. White plain, New York 10602, USA [71]. The participant was seated with a straight back leaning against the back of a chair, wearing the inclinometer over the head, and was asked to perform active flexion, extension, rotation (left and right), and side bending (left and right) [72]—the participants were instructed to stop at the point where pain symptoms began, or otherwise to continue to the fullest extent of their mobility. Each movement was recorded three times; the lowest reading was discarded, and the mean was calculated between the two [72].

### 2.13. Intervention

The study consists of a total of 4sessions of mobilization twice a week with movement as Mulligan described.

All subjects received the same manual therapy: treatment mobilization with Mulligan movement indicated increased cervical ROM but without producing pain [18]. The procedure to perform Mulligan on the patients was carried out using 3 series of 10 repetitions of mobilizations, always taking into account the principle of performing the movement without the patient experiencing pain during the execution while the physiotherapist maintains an overpressure on the cervical region. All subjects underwent the first treatment session, and before the second, third, and fourth sessions, they were given a GRoC [57]. If the subjects marked “much better” or “so much better” (punctuation over 5 points), then they were classified as having a satisfactory result and no more treatments were performed. If the patient was not classified as successful, then the treatment from the first session was repeated and so on up to a maximum of 4 sessions. Just after the fourth session, a final assessment was performed. The cervical level treated was different for each patient, because each patient had symptoms at a different level or on a different side, and the treatment was applied to the site where the patient had relief of symptoms when moving the neck.

### 2.14. Statistical Analysis

Statistical analysis was carried out using the R program Ver. 3.5.1. (R Foundation for Statistical Computing, Institute for Statistics and Mathematics, Welthandelsplatz 1, 1020 Vienna, Austria). The significance level was set at *p* < 0.05. Missing values were treated with multiple imputations using the predictive mean matching (PMM) method, as the percentage of missing data did not exceed 5% (Supplementary Materials Table S1). The distribution of quantitative variables was tested using the Kolmogorov–Smirnov test with Lilliefors correction (Supplementary Materials Table S2). Qualitative variables were described in terms of absolute values and frequencies, and quantitative variables were described in terms of mean and standard deviation.

#### Machine Learning Analysis

The sample size was adjusted using the one in five rule for the initial model [73] and one in ten for the final model [74], that is, five and ten subjects per explanatory variable. A logistic regression model was applied between the binary dependent variable GRoC (success or no success) and the explanatory variables of age, chronicity, gender, body mass index, number of treatments, pain catastrophizing scale (ECD), Tampa scale of kinesiophobia (TSK-11), Beck Depression Inventory (BDI II), State-Trait Anxiety Inventory (STAI), Neck Disability Index (NDI), SF12 physical status, SF12 mental status, VAS, flexo-extension ROM, side bending ROM, and rotation ROM. The backward stepwise method was used to select the model with the lowest AIC (Akaike information criterion) after eliminating variables with a VIF (variance inflation factor) higher than 5. The resulting model was compared with the saturated model of all variables using an ANOVA table to select the final model. From the final model, the coefficients and odds ratio were calculated with the corresponding confidence intervals (95% CI) as well as the level of significance. The final model was evaluated by bootstrap calibration. The goodness of fit was determined using the X^2^ and Hosmer–Lemeshow statistics. A sensitivity analysis was carried out using the receiver operating curve (ROC), determining the sensitivity, specificity, and AUC (area under curve), taking as cut-off values those above 0.5. The explained variability was calculated with Nagelkerke’s pseudo R^2^. An analysis of the outliers and influential values as well as residuals was performed, Classification techniques based on machine learning were also applied to the final model: logistic regression which modeled the probability of an event taking place by setting the log-odds for the event as a linear combination of one or more independent variables, classifications, and decision trees, both supervised learning techniques that have a pre-defined target: random forest, which is an ensemble learning method for classification, regression, and other tasks that operates by constructing a multitude of decision trees at training time; Generalized Boosted Models (GBM), which repeatedly fit many decision trees to improve the accuracy of the model; Support Vector Machines (SVM) with basic linear and radial kernel which are based on pattern analysis; and neural networks, which analyze data based on a collection of connected units or nodes called artificial neurons (in the latter, the number of neurons was selected according to the lowest Standardized Root Mean Square Error [SRMSE]). The AUC, sensitivity, specificity, and accuracy of these models were evaluated when applied to new data, as well as the importance of the variables contained in them.

## 3. Results

### 3.1. Baseline Results

A total of 80 subjects between 18 and 60 years of age were included (32.70 ± 12.79 years), and there was a higher number of females than males 68.8% vs. 31.2% (Table 1).

**Figure 1:**
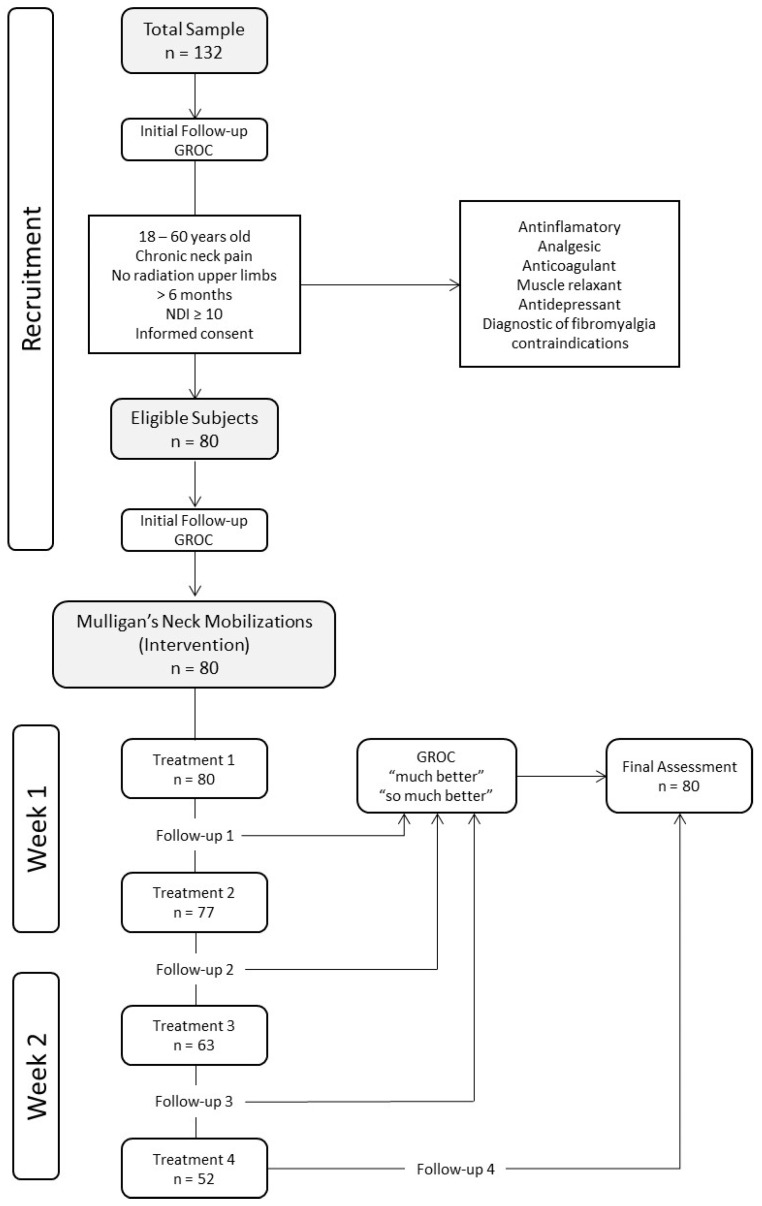
Flow chart of the study.

Of the total number of patients, only 3 patients received a single session, 77 received 2 sessions, 63 received 3 sessions, and 52 received 4 sessions (Figure 1).

### 3.2. Main Outcomes

**Figure 2:**
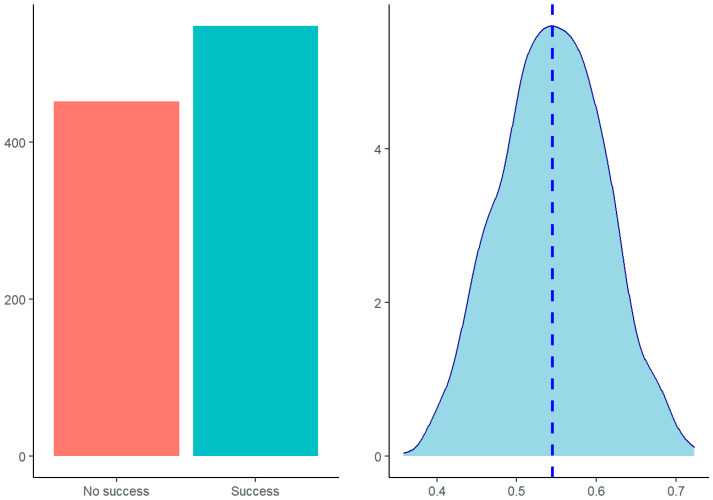
Model predictions (left) and predictions’ density distribution (right).

The final model selected contained the variables of the State-Trait Anxiety Inventory (STAI) and the left–right side bending ROM, which predicted success/non-success significantly. The model predictions showed a higher probability of success (54.8% vs. 45.2%) (Figure 2). 

The odds ratios indicated that for each of increase in the left–right side bending ROM, the probability of success decreased 0.968 times, while for each point of increase in the State-Trait Anxiety Inventory (STAI), the probability of success increased 1.083 times (Table 2).

### 3.3. Model Quality

The bootstrap calibration corroborated the overall significance of the model (X^2^(2) = 12, *p* = 0.002) with high discrimination ranks above 0.6 (AUC = 0.698), similar to corrected AUC = 0.676, indicating no overestimation of the model. The ratio deviancy/residual deviancy was 1.276, less than 1.5, indicating that the model was not over dispersed.

The model’s deviancy of 98.26 (77) was lower than the null of 110.7 (79), and its significant effectiveness (X^2^(2) = 12.444, *p* = 0.002), indicated that the model significantly predicted success/non-success. The non-significant Hosmer–Lemeshow test (*p* = 0.112) corroborated the above. Residual plots showed an adequate model fit and the absence of influential values.

**Figure 3:**
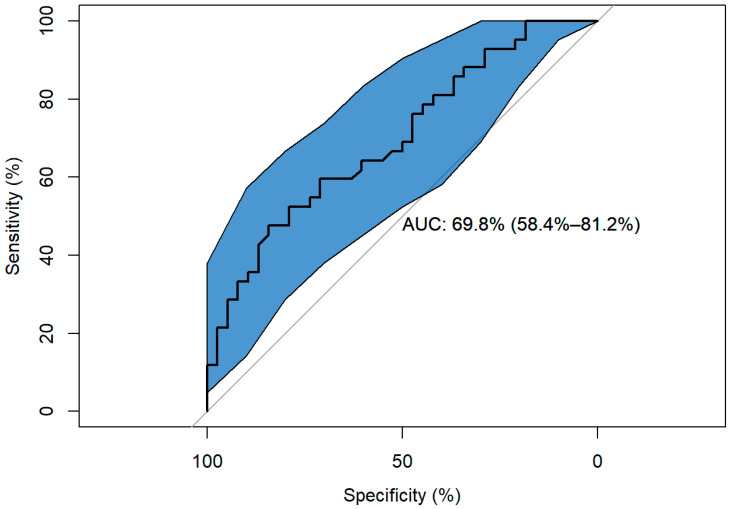
Model ROC curve.

Sensitivity and specificity were around 65.288% with a cut-off point of 55.657%. The ROC curve showed a significant AUC of 69.768%, 95%CI (58.357%–81.18%) (Figure 3). The classification table indicated that, with a cut-off point of 0.5, the model correctly classified 37.5% of the subjects, which was accompanied by a low Nagelkerke’s pseudo R^2^ = 0.192.

The ML models with the highest predictive ability were the generalized boosted model, logistic regression, support vector machine, and linear kernel support vector machine, with an AUC of 0.850, 0.694, 0.694, and 0.694, respectively (Table 3). The most important variable in predicting success/non-success was the left–right side bending ROM (Table 4).

## 4. Discussion

The aim of this study was to develop a predictive ML approach in order to determine which variables have the most influence on the improvement of patients with neck pain who undergo physical therapy treatment based on the Mulligan concept.

The ML models with the highest predictive ability were the generalized boosted model, logistic regression, support vector machine, and linear kernel support vector machine. The novelty of this study is the development of a data-driven model, such as ML [75]. In the data-driven model, the predictors are adjusted according to the outcome in order to achieve the best predictive accuracy of the model [75]. In the present study, analysis was not performed with regression coefficients, because classical techniques do not take into account the probabilistic nature of the predictors and the outcome, whereas this is inherent to ML [76].

The performance of our models was comparable to previous ML prediction models developed for low back pain [77] and a more recent one for patients with cervical radiculopathy [28], as the predictive accuracy of the modelling methods is clinically very similar.

Few published studies of prognostic modelling in musculoskeletal pain have considered a non-linear relationship in order to more accurately assess the non-linear relationship in chronic pain processes [78].

In the final model, two important predictors were identified: left–right side bending ROM and anxiety. Neck pain patients with lower lateroflexion ROM and higher anxiety were more likely to succeed with Mulligan treatment. The results of the present study are contrary to those obtained by Snodgrass et al. [79] who found limited evidence to suggest that restricted cervical ROM is associated with negative outcomes of manual therapy and that greater ROM is associated with positive outcomes. These results must be taken with caution, as the studies were very heterogeneous in terms of population, manual therapy technique used, and the plane of restricted ROM. Puentedura et al. [64]. developed a clinical prediction rule to identify patients with neck pain who would benefit from cervical spine manipulation, and one of the predictors present was a side-to-side difference in cervical rotation range of motion of 10° or greater. In contrast, Saavedra-Hernandez et al. [80] found cervical extension less than 46° to be one of the prognostic factors. These results must be taken with caution, as the studies were very heterogeneous in terms of population, manual therapy technique used, and the plane of restricted ROM. In the current study, Mulligan mobilization was used instead of manipulation, and the predictor was latero-flexion. This suggests that neck pain patients with more-limited cervical ROM may benefit more from manual therapy, but further studies are needed to clarify which planes of ROM are the best predictors.

The presence of anxiety may cause the patient to be more receptive to receiving the technique and have higher expectations of success, therefore it may have nocebo effects on the patient [81]. However, A recent study of students with neck pain found that increased anxiety was associated with increased neck disability among university students [82]. In two previous studies [83,84] in which a clinical prediction rule was made when applying manual therapy and exercises in patients with non-specific chronic neck pain, they have found that fear-avoidance influences were a psychological variable in the predictive models they had developed, but they did not find anxiety as happened in the present study. This is mainly because in those studies the anxiety variable was not included.

The literature reports that patients with neck pain have an association between neck pain severity and ROM restrictions, disability, and psychological variables, and that these variables are predictors of chronicity [85,86]. For this reason, since we found greater improvement in these patients in the present study, the results obtained are encouraging and suggest a greater therapeutic effect in patients with less ROM and high levels of anxiety. 

### 4.1. Future Research Directions

Future prognostic research will be necessary to improve the accuracy of treatments in the recovery of patients based on the predictors found in this study. For example, studies combining manual therapy techniques with therapeutic exercise interventions and different dosages must be conducted in order to improve decision-making in the clinical setting. The solid methods used by ML could be a crucial step in future directions. Another line of future research—after the first step of internal validation of the prognostic model—could be an external one [35], using data from RCTs and including other physical therapy approaches targeting chronic neck pain or investigating the impact of the model in clinical practice before implementation [35].

### 4.2. Limitations

The current study had multiple limitations. The first is the sample size, in terms of the number of variables included when developing the model [35]. Second, for outcome variables, it is difficult to judge to what degree the results are generalizable to other settings. To avoid this limitation, the predictive values chosen were selected on a clinical basis and in consensus with the researchers. Selecting the outcomes for the pre-selection approach, the authors of the present study were not blinded to the effects of the single factors. The prognostic capacity of subgrouping staging (also in combination with other factors) had been investigated previously, indicating low capacity. Third, it should be noted that the sample was supposed to be heterogeneous with respect to the age of the patients, since the inclusion and exclusion criteria included patients between 18 and 60 years of age who presented chronic neck pain. However, finally the mean and standard deviation of 32.70 ± 12.79 minimized the heterogeneity. Noting that musculoskeletal structures’ recovery, sensation, or behavior is not the same in young subjects as in older ones, we finally avoided the wide age range of the sample which could be a limitation considering the differential levels of regional brain activations [87,88]. However, as a strength, age was included as a covariate in all analyses. Furthermore, other structures’ pain should be studied widely to generalize these results. The predictive power of models was not evaluated in unseen data, and the outcome was obtained just after treatment, which could implicate in bias.

Finally, the predictive ability of our model was well-designed and valid, but there might be other factors that could help predict the outcome more accurately, such as, for example, the consideration of lifestyle factors and psychological ones related to work, or tobacco, alcohol, or drug consumption [89]. It is necessary to take into account that our predictive model could make clinicians consider the important factors to predict which patients will have the best success in a clinical setting. These factors can vary depending on the outcome measured, and the chance of improvement may change over time for some factors [85].

## 5. Conclusions

Anxiety and ROM for lateroflexion basal outcomes have been shown to indicate a good prognostic value for Mulligan’s mobilization technique. The most important predictive factors for recovery in chronic neck pain patients indicated that the more anxiety and the lower the ROM of lateroflexion, the higher the probability of success with the Mulligan concept treatment. Meanwhile, the greater the range of movement of lateroflexion and the lower the anxiety of the patients, the lower the probability of success of treatment using Mulligan techniques.

## Figures and Tables

**Table 1:** Clinical and demographic characteristics of the participants.

Characteristics		n = 80
Age		32.70 ± 12.79
Chronicity		43.24 ± 40.11
Body Mass Index		22.70 ± 3.20
Pain Catastrophizing Scale		8.47 ± 6.71
Tampa Scale of Kinesiofobia-11		23.29 ± 5.04
Beck’s Depression Inventory		27.60 ± 5.21
State-Trait Anxiety Inventory		17.99 ± 7.29
Neck Disability Index		10.14 ± 4.75
SF12 Physical status		46.67 ± 8.63
SF12 Mental status		48.09 ± 8.71
VAS		2.56 ± 1.46
Flexo-extension ROM		129.45 ± 28.03
Left-right side bending ROM		94.29 ± 23.09
Left-right rotation ROM		151.10 ± 28.70
GRoC, n (%)	No success	38 (47.5)
	Success	42 (52.5)
Gender, n (%)	Female	55 (68.8)
	Male	25 (31.2)

VAS: visual analog scale; ROM: range of motion; SF-12: health questionnaire short Form-12. Data were expressed as the mean + standard deviation or with absolute and relative values (%).

**Table 2:** Final model summary.

	Odds Ratio 95% CI	Coefficient ± SE	95%CI	Z Value	^a^*p* Value
(Intercept)	5.822 (0.574, 72.158)	1.762 ± 1.218	−0.556, 4.279	1.447	0.148
State-Trait Anxiety Inventory	1.083 (1.012, 1.171)	0.08 ± 0.037	0.012, 0.158	2.178	0.029
Left-Right Side Bending ROM	0.968 (0.943, 0.99)	−0.032 ± 0.012	−0.058, −0.01	−2.668	0.008

SE: standard error. ROM: range of motion. ^a^ significant if *p* < 0.05.

**Table 3:** Model quality.

	F1 Score	AUC	Sensitivity	Specificity	Accuracy (95% CI)	Accuracy (*p*-Value)	Kappa	McNemar (*p*-Value)
Generalized Boosted Model	0.857	0.850	0.7	1.000	0.842 (0.604, 0.966)	0.004	0.689	0.248
Logistic Regression	0.727	0.694	0.5	0.889	0.684 (0.434, 0.874)	0.125	0.380	0.221
Support Vector Machine	0.727	0.694	0.5	0.889	0.684 (0.434, 0.874)	0.125	0.380	0.221
Linear Kernel Support Vector Machine	0.727	0.694	0.5	0.889	0.684 (0.434, 0.874)	0.125	0.380	0.221
Neural Network	0.727	0.694	0.5	0.889	0.684 (0.434, 0.874)	0.125	0.380	0.221
Classification Tree	0.640	0.544	0.2	0.889	0.526 (0.289, 0.756)	0.592	0.086	0.046
Radial Basis Function Kernel	0.583	0.489	0.2	0.778	0.474 (0.244, 0.711)	0.755	−0.022	0.114
Random Forest	0.421	0.578	0.6	0.556	0.421 (0.203, 0.665)	0.875	−0.155	1.000
Decision Tree	0.154	0.406	0.7	0.111	0.421 (0.203, 0.665)	0.875	−0.194	0.228

AUC: area under the curve; 95% IC: 95% confidence interval. Significant if *p* < 0.05.

**Table 4:** Importance variables in best models.

	Generalized Boosted Model	Logistic Regression	Support Vector Machine
Left-Right Side Bending ROM	100.00	100.00	100.00
State-Trait Anxiety Inventory	0.00	0.00	0.00

ROM: Range of Motion. Data expressed with absolute and relative values (%).

## Data Availability

Data available on request due to privacy and ethical restrictions.

## Supplementary Materials

The following supporting information can be downloaded at: https://www.mdpi.com/article/10.3390/life13010048/s1, Table S1: Missing data. Table S2: Distribution of quantitative variables.
