# Supplementary material for: Prediction of Patient Satisfaction after Treatment of Chronic Neck Pain with Mulligan’s Mobilization
Source: Life (Basel). 2022 Dec 23;13(1):48. doi: 10.3390/life13010048 (PMC9860852; doi:10.3390/life13010048)
Supplement: Supplementary file 1 [file life-13-00048-s001.zip › life-2071494-supplementary.pdf]

Supplementary material. Table S1: Missing data.

GROC (sucess/ no sucess)	3 (0.51%)	State-Trait Anxiety Inventory	4 (0.68%)
Age	2 (0.34%)	Neck Disability Index	1 (0.17%)
Chronicity	2 (0.34%)	SF12 Physical status	2 (0.34%)
Gender	2 (0.34%)	SF12 Mental status	2 (0.34%)
Body Mass Index	2 (0.34%)	VAS	1 (0.17%)
Number of treatments	0 (0%)	Flexo-extension ROM	6 (1.02%)
Pain Catastrophizing Scale	4 (0.68%)	Left-right side bending ROM	6 (1.02%)
Tampa Scale of Kinesiofobia-11	4 (0.68%)	Left-right rotation ROM	6 (1.02%)
Beck's Depression Inventory	4 (0.68%)		

Supplementary material. Table S2: Distribution of quantitative variables.

	W	[a] p value		W	[a] p value
Age	0.203	<0.001	Neck Disability Index	0.119	0.007
Chronicity	0.225	<0.001	SF12 Physical status	0.137	0.001
Body Mass Index	0.084	0.176	SF12 Mental status	0.118	0.008
Number of treatments	0.171	<0.001	VAS	0.136	0.001
Pain Catastrophizing Scale	0.145	<0.001	Flexo-extension ROM	0.108	0.022
Tampa Scale of Kinesiofobia-11	0.133	0.001	Left-right side bending ROM	0.093	0.082
Beck's Depression Inventory	0.131	0.002	Left-right rotation ROM	0.135	0.001
State-Trait Anxiety Inventory	0.072	0.376			

[a] no-normal distribution if p<0.05.
